# EPSPS target site mechanisms confer glyphosate resistance in *Arctotheca calendula*

**DOI:** 10.1002/ps.8690

**Published:** 2025-02-05

**Authors:** Norazua Zakaria, Heping Han, Yaseen Khalil, Michael Ashworth, Qin Yu, Ken C Flower

**Affiliations:** ^1^ Department of Crop Science, Faculty of Agriculture Universiti Putra Malaysia Serdang Malaysia; ^2^ Australian Herbicide Resistance Initiative, School of Agriculture & Environment University of Western Australia Perth Western Australia Australia; ^3^ Kalyx Australia Pty Ltd Kewdale Western Australia Australia

**Keywords:** capeweed, glyphosate‐resistance, target‐site resistance, EPSPS

## Abstract

**BACKGROUND:**

The first case of glyphosate resistance was reported in a capeweed population from Western Australia in our previous study. This current study investigates the resistance mechanisms in the population.

**RESULTS:**

Target‐site *EPSPS* gene sequencing revealed two partial sequences of the *EPSPS* transcripts (1001 bp and 998 bp), and the full‐length sequence (1551 bp) containing the 1001‐bp transcript was cloned as it was found in the resistant plants. A known resistance‐endowing target‐site mutation in the 1551‐bp transcript was identified in the resistant plants, resulting in the Pro‐106‐Ser substitution. The subpopulation derived from these mutant plants exhibited >10‐fold resistance to glyphosate compared to the susceptible population. Additionally, the *EPSPS* gene (1551 bp) was constitutively expressed at a higher level (4.3‐fold) in the resistant than in the susceptible populations. However, ^14^C‐glyphosate foliar uptake was similar with no visual difference in ^14^C‐glyphosate translocation from leaves to other parts of a plant, between the resistant and susceptible population.

**CONCLUSION:**

Glyphosate resistance in the studied population is associated with both a target‐site mutation (Pro‐106‐Ser) and increased *EPSPS* gene expression. © 2025 The Author(s). *Pest Management Science* published by John Wiley & Sons Ltd on behalf of Society of Chemical Industry.

## Graphical abstract

This investigation revealed that the *EPSPS* gene mutation resulting in Pro‐106‐Ser substitution and *EPSPS* overexpression endow resistance to glyphosate in the capeweed population.

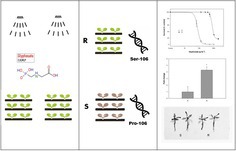

## 1 INTRODUCTION

Glyphosate is the world's most important herbicide, and its sustainability is threatened by the evolution of resistance in weeds. Glyphosate resistance was first reported in rigid ryegrass (*Lolium rigidum*) in 1996,^1^ and since then the evolution of glyphosate‐resistant weed populations has rapidly increased.^2^, ^3^, ^4^ Presently, glyphosate resistance has been identified in populations of 59 weed species across 32 countries.^4^

Glyphosate causes plant death by depriving them of essential aromatic amino acids. The 5‐enolpyruvylshikimate‐3‐phosphate synthase (*EPSPS*; EC. 2.5.1.19) is responsible for biosynthesis of aromatic amino acids phenylalanine, tyrosine and tryptophan.^5^ Given the prevalence and significant implications of glyphosate‐resistant (GR) weed evolution, there has been an intensive research effort to understand the biochemical and molecular basis of the resistance.

Like resistance to other herbicides, glyphosate resistance involves target‐site and nontarget‐site mechanisms.^6^ Target‐site resistance resulting from *EPSPS* mutations has been well‐documented including *EPSPS* single mutations at Pro‐106,^7^ Thr‐102^8^ and Ala‐100,^9^ double mutations at Thr‐102‐Ile + Pro‐106‐Ser (TIPS)^10^ and Thr‐102‐Ile + Pro‐106‐Thr (TIPT),^11^ and the triple mutation Thr‐102‐Ile + Ala‐103‐Val + Pro‐106‐Ser (TAP‐IVS).^12^, ^13^ Additionally, EPSPS gene copy number variation (CNV) has been identified as a more common target‐site resistance mechanism in many weed species.^14^, ^15^ Contrariwise, nontarget‐site glyphosate resistance involves reduced glyphosate foliar uptake and/or translocation, increased glyphosate vacuole sequestration or extracellular extrusion.^16^ Additionally, genes for glyphosate metabolism by aldo‐keto reductases (AKRs) and for glyphosate extrusion by an ABC transporter (ABCC8) recently have been cloned and characterized in *Echinochloa colona*.^17^, ^18^

Capeweed (*Arctotheca calendula*) is a widespread dicot weed that infests crops and pastures in Australia.^19^, ^20^ It causes crop yield losses even at low densities. For instance, 7–90 capeweed plants m^−2^ can reduce grain yield by 28% to 44%.^21^ Furthermore, the evolution of herbicide resistance to diquat and 2,4‐D has been reported in capeweed.^4^, ^22^ Recently we reported the first case of glyphosate resistance in capeweed from Western Australia, and preliminary studies indicated nontarget‐site resistance mechanisms to glyphosate.^23^ This current study investigates nontarget‐site glyphosate foliar uptake and translocation, and re‐examines target‐site EPSPS mutation and other possible target‐site mechanisms. Results showed that capeweed has at least two *EPSPS* transcripts although it is a diploid species, and both EPSPS target‐site mutation (Pro‐106‐Ser) and higher *EPSPS* expression contribute to glyphosate resistance in the capeweed population studied.

## 2 MATERIALS AND METHODS

### 2.1 Plant materials

The glyphosate‐susceptible (S) and ‐resistant (R) populations of capeweed used in this current study were from our previous study.^23^ Seeds were germinated in plastic trays (300 mm × 350 mm) containing potting mix (50% fine composted pine‐bark, 20% coco peat, 30% river sand) in a temperature‐controlled glasshouse (20/18 °C day/night) under natural sunlight. Germinating seedlings were transplanted at the two‐ to four‐leaf stage, and were foliar treated with 540 and 1080 g glyphosate ha^−1^ (Roundup PowerMax, 540 g a.e. L^−1^ Glyphosate, Nufarm, Australia), using a custom‐built cabinet sprayer delivering a volume of 107 L ha^−1^ at 200 kPa with a speed of 1 m s^−1^. Three weeks after treatment, seven best‐surviving plants from the R population (with active new growth) were identified and transplanted for the following experiments.

### 2.2 *EPSPS* gene sequencing

Total RNA was isolated from the leaf tissue of the seven individual R and five bulked S plants using the Isolate II RNA plant Kit (Bioline, Memphis, TN, USA). Genomic DNA contamination was removed using the TURBO DNA‐free kit (Ambion/Thermo Fisher Scientific, Waltham, MA, USA). Complementary DNA (cDNA) was synthesized from 2 μg RNA using the SuperScript III reverse transcriptase (Invitrogen/ Thermo Fisher Scientific). The forward primer, TrEPSPSF1 (5′‐AAGTCTTTGTCTAATCGGAT‐3′) and the reverse primer, TrEPSPSR1 (5′‐CAGCTAGCCACGTCTCTAATG‐3′),^8^ were used to amplify EPSPS fragments covering the known mutation sites. The PCR was conducted in a 25‐μL volume including 50–100 ng cDNA, 0.4 μm each primer, 200 μm each of dNTP mixture, 5 μL 5× PrimeSTAR GXL buffer and 0.6 U PrimeSTAR GXL polymerase (TaKaRaBio, Tokyo, China). The PCR was run with the following profile: 40 cycles of 98°C for 30 s, 55°C (annealing temperature) for 15 s and 68 °C for 70 s. The amplified cDNA fragment was cloned into the pGEM‐T vector (Promega, Madison, WI, USA) and transformed into JM109 competent *Escherichia coli* cells (Promega). Plasmids from white colonies containing the right insert were extracted with a Wizard plus SV Minipreps DNA Purification System (Promega) and sequenced by commercial services. All chromatograms were examined visually to ensure their quality and consistency and compared between S and R samples.

### 2.3 Generation of the purified R subpopulation and dose–response to glyphosate

The sequencing results revealed that all the seven individual R plants possess the single *EPSPS* Pro‐106‐Ser mutation. Therefore, these seven plants were maintained in the glasshouse in isolation, grown to maturity, and hand‐crossed to generate the R subpopulation for the herbicide dose response experiment. Seeds of the purified R subpopulation and the S population were germinated and transplanted as above in plastic trays (20 seedlings per tray). At the four‐ to six‐leaf stage, glyphosate was applied at 0, 67.5, 135, 270, 540, 1080 g ha^−1^ for S plants, and 0, 135, 270, 405, 540, 1080, 2160 g ha^−1^ for R plants. Three weeks after treatment, plant mortality was assessed, with plants that had no new growth being recorded as dead. Each treatment contained two to three replicate trays.

### 2.4 Full *EPSPS* cDNA cloning

Total RNA was isolated from leaf tissue of individual R and S plants, using the TRIzol™ (Invitrogen, Carlsbad, CA, USA) according to the manufacturer's instructions with some modifications. Genomic DNA contamination was removed using the TURBO DNA‐free kit (Ambion). The 5′‐ and 3′‐RACE System for rapid amplification of cDNA Ends (Invitrogen) was used for cloning the *EPSPS* 5′‐ and 3′‐cDNA flanking sequences. Three gene‐specific reverse primers, AcEPSPS765R (5′‐CCCACAGCCTTCCACCG‐3′), AcEPSPS251R (5′‐CGCATAGCAGTTCCCGC) and AcEPSPS204R (5′‐ACTAGCTTCTCTACCCACTGGA‐3′), and two forward primers, AcEPSPS631F (5′‐CGGTTTTACGTCAAAGGTGGT‐3′) and AcEPSPS735F (5′‐TGGCGGAACCATCACGG‐3′), were designed to generate 5′‐RACE and 3′‐RACE cDNA fragments. The PCR was conducted in a 25‐μL volume as described above. The PCR was run with the following profile: 35 cycles of 98°C for 30 s, 55°C (annealing temperature) for 15 s and 68 °C for 70 s. The PCR product was purified from the agarose gel using the Wizard SV Gel and PCR Clean‐Up System (Promega). The amplified PCR fragment was cloned into the pGEM‐T vector (Promega) and transformed into competent *E. coli* cells. The putative inserts were sequenced by commercial services and sequences chromatograms were visually checked for quality and clarity.

Based on fully assembled *EPSPS* cDNA sequences, the forward primer, AcEPSPS Full F (5′‐ATG GCG GTT CAC GTT AAC AAC‐3′) and reverse primer, AcEPSPS Full R (5′‐TTA ATG CTT GGT GAA TCT TTC AAG‐3′), were designed to amplify the full *EPSPS* cDNA sequences from S and R plants and compared to the assembled ones.

### 2.5 *EPSPS* gene expression

Total RNA was isolated from the three‐ to four‐ leaf stage plants of the S (three individuals) and R (5 individuals) populations using the TRIzol™ (Invitrogen) and quantified using a Nanodrop 2000 spectrophotometer (Thermo Fisher Scientific) before quantitative reverse transcription (qRT)‐PCR analysis. For measuring *EPSPS* gene expression, the forward primer AcEPSPSF (5′‐CTGGTGGCAAGGTCAAATTATC‐3′) and reverse primer AcEPSPSR (5′‐ TTCGATTTCCACGTCTCCTAAC‐3′) were designed based on the sequenced capeweed R *EPSPS* allele (1001 bp) in this study, to amplify a 150‐bp cDNA fragment. The forward primer, AcTubulinF (5′‐TCG TGG AGA TGT TGT TCC TAA AG‐3′) and reverse primer, AcTubulinR (5′‐GAC TGT TGG TGG TTG GTA GTT‐3′), were used to amplify a 98‐bp *α‐tubulin* cDNA fragment as a reference, based on the known *α‐tubulin* sequence from *E*. *colona*.^24^ The qRT‐PCR system containing 50 ng cDNA, each primer at 0.125 μm, and 10 μL of 2× SYBR Green mix in a total volume of 20 μL, was run using the following standard procedure from 7500 Software (Life Technologies Inc., Carlsbad, CA, USA): 20 s at 50 °C, 10 min at 95 °C, 40 cycles of 95 °C for 15 s and 60 °C for 1 min; then, the temperature was gradually increased (by 0.5 °C every 5 s) to 95 °C for the generation of melting curves. Melting‐curve analysis of the PCR products confirmed the specificity of the *EPSPS* and α‐tubulin amplicon. The primer efficiency was 102% and 98% for the *EPSPS* and *tubulin* genes, respectively. Each assay included three individual (S plants) or five (R plants) biological replicates and two technical replicates. The gene expression level was expressed as 2^−△△CT^, where ∆∆CT = ∆CT (EPSPS gene of the S samples – the reference gene) – ∆CT (EPSPS gene of the R samples – the reference gene) and was subject to the Student's *t*‐test (*P* < 0.05).

### 2.6 ^14^C‐glyphosate uptake and translocation

Germinated seedlings of the S and R populations were transplanted into plastic cups (60 × 60 × 100 mm, one seedling per cup) filled with the potting mix. Seedlings were grown and maintained in a controlled environment room at alternating temperatures of 25/20°C day/night, a 12 h:12 h, light:dark photoperiod, 650 μmol m^−2^ s^−1^ light intensity and 75% relative humidity.

At the four‐leaf stage, a 1‐μL droplet of ^14^C‐glyphosate treatment solution was applied to the midpoint of the fully expanded leaf (the first pair of opposite leaves). The radiolabelled glyphosate treatment solution was prepared by diluting ^14^C‐glyphosate (glycine‐2‐^14^C, ARC, MO, USA) in commercial glyphosate formulation (Roundup PowerMax, 540 g L^−1^ Glyphosate; Nufarm, Australia) with a final concentration and 0.80 kBq μL^−1 14^C‐glyphosate plus 0.25% (v/v) nonionic surfactant BS1000, equivalent to the glyphosate rate of 270 g ha^−1^, a discriminating rate for the S and R populations.

Seven treated plants from each S and R population were harvested 24, 48 and 72 h after treatment (HAT). The treated leaves of each plant were rinsed in 20 mL washing buffer with 20% (v/v) methanol and 0.2% (v/v) Triton X‐100 to remove unabsorbed ^14^C‐glyphosate. The radioactivity present in the rinse solution was quantified by a liquid scintillation counter (Packard 1500, Tri‐carb®; Perkin Elmer, Waltham, MA, USA). ^14^C‐glyphosate leaf uptake was expressed as a percentage of the total applied. Likewise, the roots of each plant were washed in 50 mL washing buffer, and the radioactivity in the wash‐off was quantified. Afterwards, the plant samples were blot‐dried between paper towels, pressed, and then oven‐dried for 3 days at 60 °C. Movement of ^14^C‐glyphosate from the application point to other parts of the plant was visualized using Typhoon phosphor imaging (GE Healthcare, Chicago, IL, USA).

### 2.7 Statistical analysis

The herbicide rate causing 50% plant mortality (LD_50_) was statistically analysed by nonlinear regression using the four‐parameter logistic model *y* = *C* + (*D* − *C*)/[1 + (*X*/I50)^*b*^], where: *D* is the upper limit, close to the values of the untreated controls; *C* is the lower limit, close to the values from infinitely large herbicide rates; and *b* is the slope of the best‐fitting curve of LD_50_. The estimates were obtained using sigmaplot software (v12.3; Systat Software, Inc., Chicago, IL, USA). Significant differences in the datasets of glyphosate foliar uptake and gene expression between the S and R populations was analyzed by Student's *t*‐test using the SAS software (SAS, v9.4; SAS Institute Inc., Cary, NC, USA).

## 3 RESULTS

### 3.1 *EPSPS* gene sequencing

A total of 22 clones (three clones from each of the seven purified R plants and one from bulked S samples) were sequenced. Clones from the R plants contained a 1001‐bp *EPSPS* fragment (GenBank accession no. PQ838655), whereas clones from the bulked S samples contained the 1001‐bp and an additional 998‐bp fragments (GenBank accession no. PQ838656). These two fragments, covering all known resistant mutation sites, showed 85% and 93.4% identity at the nucleotide and amino acid levels, respectively, and therefore, are likely to be the two *EPSPS* alleles. As the purified R plants only had the 1001‐bp fragment, the full coding sequence of this amplicon was cloned from the R and S samples, and it has 1551 bp (GenBank accession no. PQ838654) encoding 516 amino acids. There were no SNPs between the R and S samples in the 1551‐bp sequence that cause amino acid changes except at the 106 codon where Pro‐106 in S was substituted by Ser in R plants. Therefore, the seven R plants, and the R subpopulation derived from these mutant plants, are likely to be homozygous for the 106‐Ser mutation.

### 3.2 Dose response to glyphosate

**Figure 1:**
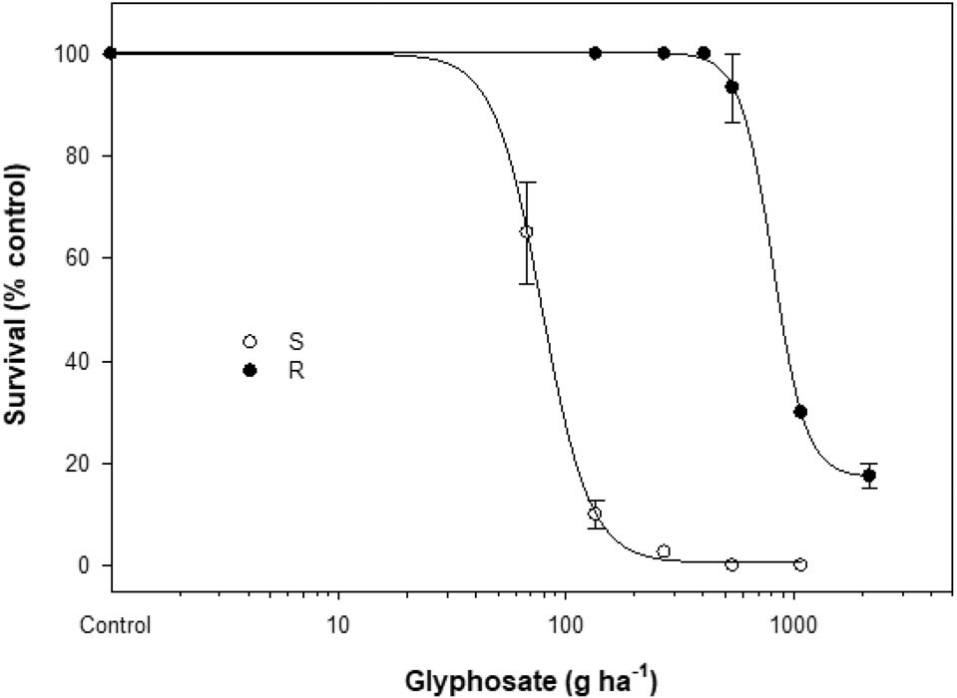
Plant mortality responses to glyphosate in susceptible (S, ○) and resistant (R, ●) capeweed populations, 3 weeks after treatment.

The level of glyphosate resistance in the purified R subpopulation was determined. The S population was controlled at glyphosate rates ≥270 g ha^−1^ (Fig. 1), with an LD_50_ value of 78.2 g glyphosate ha^−1^. By contrast, the R population showed no mortality at the recommended rate (540 g glyphosate ha^−1^), and survived the rate of 2160 g ha^−1^, with an LD_50_ value of 811 g glyphosate ha^−1^. Thus, this purified R subpopulation is 10‐fold more resistant to glyphosate than the S population (Fig. 1; Table 1).

**Table 1:** Glyphosate rates causing 50% plant mortality (LD_50_) in the resistant (R) and susceptible (S) capeweed populations, estimated by the four‐parameter log‐logistic model, and the resistance level as indicated by the LD_50_ *R*/*S* ratio

Population	*a*	*b*	LD_50_	*P*‐value	*R*/*S* ratio
*R*	100 (0.4)	17.3 (0.7)	811 (9.6)	<0.0001	10.4
*S*	100 (1.2)	0.6 (0.7)	78.3 (1.2)		

### 3.3 *EPSPS* expression

Although there are two *EPSPS* alleles (corresponding to the 1001‐ and 998‐bp fragments, respectively) in S plants, plants of the purified R subpopulation only have the mutant allele (the 1001‐bp fragment), and therefore, the qRT‐PCR primers were designed in favour of the full length of the 1001 bp (1551 bp) allele. There is a significant difference (*P* < 0.05) in the constitutive *EPSPS* expression levels between S and R samples, R being four‐fold higher than the S in expression.

### 3.4 ^14^C‐glyphosate leaf uptake and translocation

Results revealed no significant difference in foliar uptake of ^14^C‐glyphosate between S and R plants. By 24 HAT, >80% of the applied ^14^C was taken up by both the S and R leaves, a trend that persisted up to 72 h (Table 2). Glyphosate translocation from treated leaves to untreated leaves and roots increased with time, and there were no major and consistent visual differences in S and R plants in glyphosate movement (Fig. 3). Hence, it is unlikely that glyphosate resistance in the R population is related to differential leaf uptake or translocation of glyphosate.

**Table 2:** ^14^C‐glyphosate foliar uptake in the plants of glyphosate susceptible (S) and resistant (R) population at 24, 48, and 72 h after treatment

Population	Time after treatment	Uptake (% of ^14^C applied)
S	24 h	88.9 (2.2) a
R		84.4 (2.5) a
S	48 h	92.3 (1.8) a
R		92.1 (1.9) a
S	72 h	91.1 (1.4) a
R		90.7 (1.7) a

*Note*: Same letters in each R and S pair at a time point mean no significant differences by Student's *t*‐test, *P* < 0.05.

## 4 DISCUSSION

Capeweed is a cross‐pollinated, diploid species (2*n* = 18).^25^ Nevertheless, two *EPSPS* transcripts (1001‐ and 998‐bp partial sequences, respectively) were identified in the current study. These two transcripts may be two *EPSPS* copies as there was only 85% and 93.4% identity at the nucleotide and the amino acid levels, respectively (when the partial sequences were compared). Nevertheless, these two transcripts may be two alleles as only one transcript (1001 bp) that carries the Pro‐106‐Ser mutation was detected in the seven R plants best surviving glyphosate at 540–1080 g ha^−1^ (and hence its full coding sequence cloned). By contrast, in the S plants both transcripts (alleles) without the mutation were present, indicating that the selected R plants are homozygous for the mutant *EPSPS* allele. To clarify this, it would require cloning the full coding sequence of the 998‐bp transcript, as well as the DNA sequences of the two transcripts. It is noteworthy that our previous study failed to identify the *EPSPS* mutation in the original R population owing to the approach of direct sequencing which preferentially picked up the 998‐bp allele in the R plants without the Pro‐106‐Ser mutation. Re‐examination of the one available RNA sample from Khalil *et al*.^23^ by *EPSPS* cloning confirmed the presence of the two transcripts and the Pro‐106‐Ser mutation in the 1001‐bp transcript. This suggests that the previous R samples used for sequencing were likely to have been heterozygous for the mutant allele, as they were not purified against glyphosate. These findings reinforce that the negative results from herbicide target‐site gene sequencing (e.g. lack of detection of mutations) must be handled with care even in diploid species.

**Figure 2:**
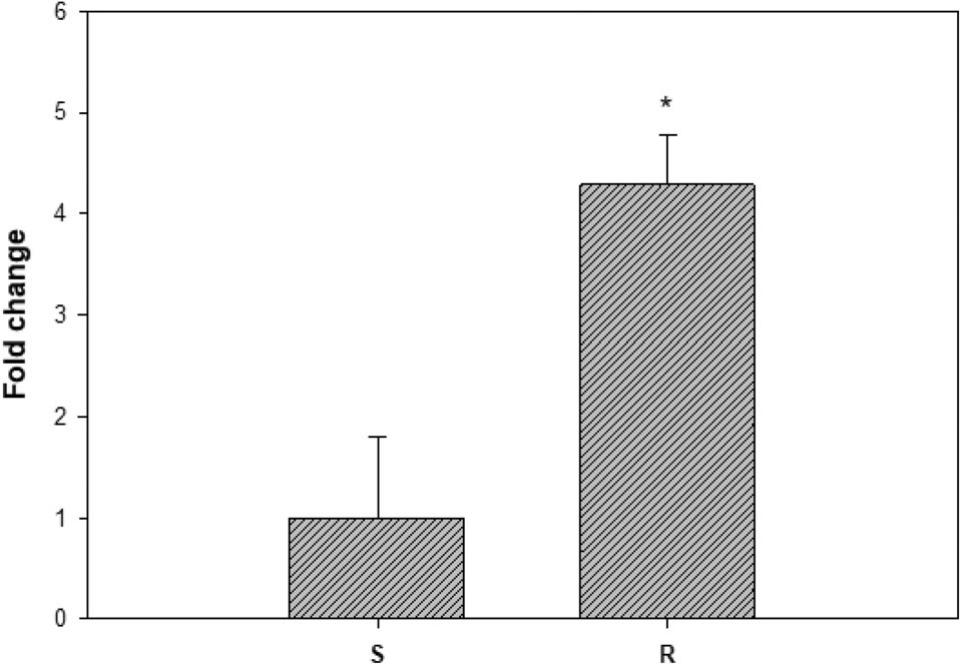
Constitutive *EPSPS* expression in the four‐leaf stage plants of glyphosate susceptible (S) and resistant (R) capeweed populations. Data are mean ± SE (*n* = 3 or 5).

Herbicide resistance level (LD_50_ or GR_50_ R:S ratio) is usually classified as high (>10), moderate (≥4–8) or low (≥2 to <3). Typically, EPSPS single mutations endow low‐to‐moderate resistance to glyphosate. However, the capeweed R subpopulation in this study displayed a 10‐fold resistance to glyphosate (Fig. 1; Table 1), indicating resistance mechanisms in addition to the identified Pro‐106‐Ser mutation. Indeed, a four‐fold higher *ESPSP* gene expression (the mutant allele) was evident in the R *versus* S plants (Fig. 2). Usually, an increase in the expression level of a herbicide target gene is correlated with copy number variation (CNV)^15^; although this remains to be determined in the R capeweed plants.

**Figure 3:**
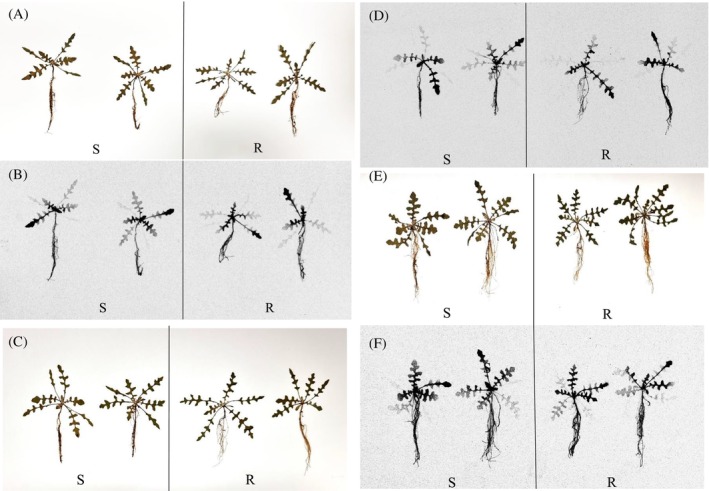
Visualization of ^14^C‐glyphosate translocation in plants of the glyphosate susceptible (S) and ‐resistant (R) capeweed populations at 24 h (A, B), 48 h (C, D) and 72 h(E, F) after treatment, with camera (A, C, E) and phosphor images (B, D, F).

In addition to target‐site resistance mechanisms, nontarget‐site reduced glyphosate translocation (or cellular glyphosate sequestration) is a common mechanism reported in many glyphosate R weed populations.^14^, ^16^, ^26^, ^27^, ^28^ In the R subpopulation, glyphosate foliar uptake and translocation was similar to that in the S capeweed population (Fig. 2), indicating the lack of alterations in glyphosate absorption and movement at the tissue level. However, extracellular or intracellular sequestration of glyphosate to the apoplast or vacuoles may be possible as has been demonstrated in glyphosate R *E. colona*,^18^ which may or may not manifested at the tissue level. Other nontarget‐site resistance mechanisms, for example glyphosate metabolism, also are a possibility as shown in *E. colona*,^17^ although not examined in the current study. Nevertheless, these possible nontarget‐site resistance mechanisms, if they do exist, may only play a minor role in glyphosate resistance in the R capeweed population investigated.

In conclusion, this investigation revealed that glyphosate resistance in the capeweed population is endowed by the Pro‐106‐Ser mutation and increased expression of the *EPSPS* gene. Glyphosate resistance, plus resistance to other herbicides in capeweed populations, necessitates effective and sustainable resistance management including the rotation and mixing of herbicide modes‐of‐action, enhancing crop competition, nonchemical weed control and incorporating cover crops.

## CONFLICT OF INTEREST STATEMENT

The authors declare no conflicts of interest associated in this article.

## Data Availability

The data that support the findings of this study are available from the corresponding author upon reasonable request.
